# NLRP3 and mTOR Reciprocally Regulate Macrophage Phagolysosome Formation and Acidification Against *Vibrio vulnificus* Infection

**DOI:** 10.3389/fcell.2020.587961

**Published:** 2020-10-08

**Authors:** Xian-Hui Huang, Yao Ma, Meng-Meng Zheng, Na Chen, Mei-Na Hu, Liu-Ying Wu, Yi Zheng, Yong-Liang Lou, Dan-Li Xie

**Affiliations:** ^1^Department of Microbiology and Immunology, School of Laboratory Medicine and Life Science, Wenzhou Medical University, Wenzhou, China; ^2^Key Laboratory of Laboratory Medicine, Ministry of Education of China, Wenzhou, China; ^3^Wenzhou Key Laboratory of Sanitary Microbiology, Wenzhou, China

**Keywords:** *Vibrio vulnificus*, NLRP3, mTOR, phagolysosome, acidification, bactericidal activity

## Abstract

The marine bacterium *Vibrio vulnificus* causes potentially fatal bloodstream infections, typically in patients with chronic liver diseases. The inflammatory response and anti-bacterial function of phagocytes are crucial for limiting bacterial infection in the human hosts. How *V. vulnificus* affects macrophages after phagocytosis is unclear. In this report, we found that the bactericidal activity of macrophages to internalize *V. vulnificus* was dependent on mammalian target of rapamycin (mTOR) and NOD-like receptor (NLR) family pyrin domain containing 3 (NLRP3) interaction. Additionally, the NLRP3 expression was dependent on mTORC1 activation. Inhibited mTORC1 or absence of NLRP3 in macrophages impaired *V. vulnificus*-induced phagosome acidification and phagolysosome formation, leading to a reduction of intracellular bacterial clearance. mTORC1 signaling overactivation could increase NLRP3 expression and restore insufficient phagosome acidification. Together, these findings indicate that the intracellular bactericidal activity of macrophages responding to *V. vulnificus* infection is tightly controlled by the crosstalk of NLRP3 and mTOR and provide critical insight into the host bactericidal activity basis of clearance of *V. vulnificus* through lyso/phagosome.

## Introduction

*Vibrio vulnificus* is a Gram-negative bacterium that causes primary septicemia, inflammation-mediated septic shock, and necrotizing wound infection (Blake et al., 1979; Park and Lee, 2018; Baker-Austin and Oliver, 2020). Cases *of V. vulnificus* infection have been reported worldwide, including the United States, China, Australia, Germany, Korea, and Japan (Zhao et al., 2015; Heng et al., 2017). Infection with this bacterium is related to *V. vulnificus* contaminated raw seafood consumption or an open wound that was exposed to warm seawater containing this bacteria (Baker-Austin and Oliver, 2020). *V. vulnificus* infection can lead to death by developing an overwhelming primary sepsis (Chou et al., 2010; Baker-Austin and Oliver, 2018, 2020). The pathogenesis of *V. vulnificus* infection, including the virulence factors, and cell death and inflammation induced by this bacteria have been well-defined (Gulig et al., 2005; Jeong and Satchell, 2012; Arezes et al., 2015; Baker-Austin and Oliver, 2018). However, little is known about the underlying mechanisms by which molecules in human hosts regulate the host’s innate immunity-mediated anti-*V. vulnificus* defense.

Phagocytes, such as neutrophils and macrophages, are the frontline cells of host defense against pathogenic microbial invasion (Russell et al., 2009; Diacovich and Gorvel, 2010). Macrophages play an essential role in the uptake and clearance of pathogens and detect harmful stimuli, including various bacterial components and infectious agents that appear during bacterial infection, *via* the receptors or phagocytosis of microbes (Ishii et al., 2008). Subsequently, macrophages are activated and produce various inflammatory cytokines, chemokines, and antimicrobial peptides. These macrophage-produced signaling molecules facilitate the activation of innate immune signaling pathways, such as transcriptional regulation signaling, inflammasome activation signaling, and mammalian target of rapamycin (mTOR) signaling (Benoit et al., 2008; Bauernfeind et al., 2009; Weichhart et al., 2015). mTOR is a serine/threonine kinase that regulates various effector responses of both innate and adaptive immune cells. mTOR forms two unique complexes: mTORC1 and mTORC2. Both complexes act as crucial regulators of multiple cellular processes in the innate immune cells, including protein translation, cytokine production, metabolism, cell survival, macrophage polarization, and self-renewal (Pan et al., 2012; Byles et al., 2013; Weichhart et al., 2015; Deng et al., 2017). Many extracellular signals can induce mTORC1 and mTORC2 activation in innate immune cells, including toll-like receptors (TLR), cytokines, and growth factors. mTOR signaling also plays a dynamic role in the regulation of macrophage activation, which further drives macrophage polarization through tight control of the cellular metabolic process (Byles et al., 2013; Covarrubias et al., 2015; Horng, 2015; Weichhart et al., 2015).

Internalization of microbes into phagosomes is a critical component of host defense. Once a macrophage internalizes the bacterium, then it can activate specific inflammasome formation, a response to cytosolic “danger signals” from the pathogens (Russell et al., 2009; Koizumi et al., 2012). The NOD-like receptor (NLR) family pyrin domain containing 3 (NLRP3) is critical for inflammasome activation. Activated caspase-1 cleaves pro-interleukin (IL)-1β to mature form IL-1β in response to microbial molecules, pyroptosis, and endogenous danger-associated molecules (Schroder et al., 2010; Anand et al., 2011; Shi et al., 2017). We previously reported that *V. vulnificus* could directly induce macrophages to produce a variety of pro-inflammatory cytokines, such as tumor necrosis factor (TNF)-α, IL-6, and IL-1β (Xie et al., 2017). Recent studies have shown that both NLRP3 inflammasome signaling and mTOR signaling in macrophages can be activated in response to bacterial cytotoxins upon *V. vulnificus* infection (Toma et al., 2010; Xie et al., 2017). Likewise, NLRP3 inflammasomes can be activated in response to other microbes, such as *Salmonella typhimurium*, *Listeria monocytogenes*, and *Staphylococcus aureus* (Munoz-Planillo et al., 2009; Kim et al., 2010; Melehani et al., 2015; Pereira et al., 2016; Qu et al., 2016; O’Neill, 2017). Even though the relationship between innate immune signaling and phagocytosis in macrophages is well-established, the molecular mechanisms of bactericidal activity by macrophages against *V. vulnificus* infection remain poorly understood.

In this report, we found that the *V. vulnificus* infection boosted NLRP3 expression and mTORC1 activation. Endogenous NLRP3 interacted with mTOR in the macrophage. Knocking out NLRP3 impaired mTORC1 signaling, interfered phagolysosome formation by suppressing phagosome acidification, and resulted in insufficient intracellular *V. vulnificus* elimination. Moreover, inhibited mTORC1 signaling suppressed *V. vulnificus*-induced NLRP3 expression and caused insufficient phagosome acidification. Besides, mTORC1 signaling overactivation could increase NLRP3 expression and restore insufficient phagosome acidification. Our study demonstrates that intracellular bactericidal activity of macrophages against *V. vulnificus* infection is tightly controlled by the novel crosstalk of NLRP3 and mTOR, and it could be a new therapeutic approach for bacterial infections. Additionally, these findings provide evidence that NLRP3 signaling and mTORC1 signaling are integrated by protein–protein interaction between the signal-transducing intracellular elements from both pathways.

## Results

### Enhanced NLRP3 Expression by *V. vulnificus* Is Required for Intracellular Bacteria Elimination

**FIGURE 1:**
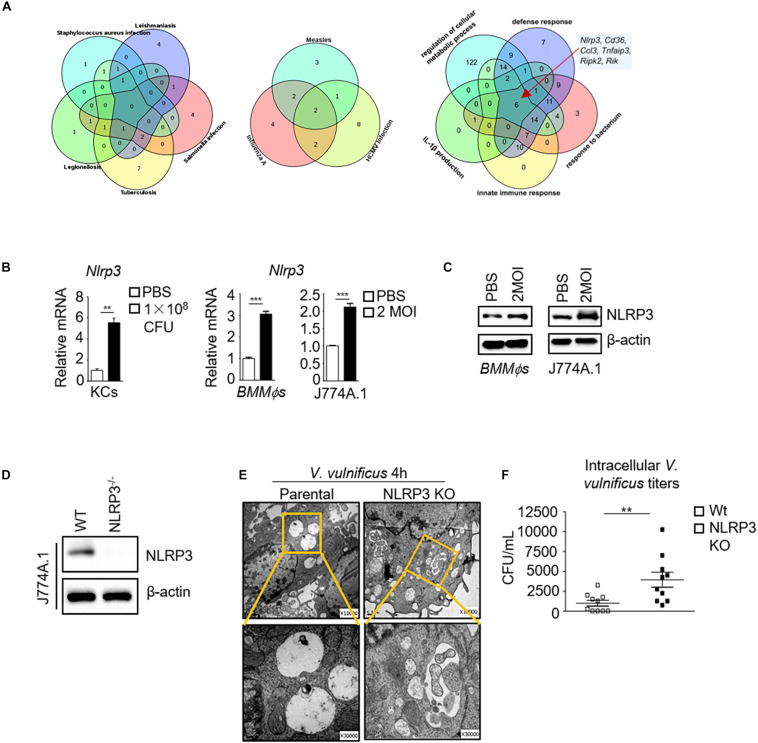
The absence of NLRP3 impairs bactericidal activity in *V. vulnificus*-infected macrophages. **(A)** RNA-Seq analysis in PBS-treated or 2 MOI *V. vulnificus*-infected J774A.1 cells for 4 h. The Venn diagrams shown are genes expressed in indicated infectious diseases (KEGG) and pathways (GO term) responding to *V. vulnificus* infection. The Venn diagram shows the overlap between each set of genes that are differentially expressed genes across indicated infectious diseases or host defense pathways (*P* < 0.05). **(B)** mRNA expression of *Nlrp3* in sorted KCs from 1 × 10^8^ CFU *V. vulnificus*-infected mice or in 2 MOI *V. vulnificus*-infected *BMMϕs* and J774A.1 cells. **(C)** NLRP3 protein expression in 2 MOI *V. vulnificus*-infected *BMMϕs* and J774A.1 cells. **(D)** Generation of an NLRP3-deficient J774A.1 cell line by CRISPR–Cas9 gene-editing system. Identification of the NLRP3 expression in biallelic deletion clones by Western blots. **(E)** Representative images of transmission electron microscopy are shown in the internalization of 2 MOI *V. vulnificus* in NLRP3-deficient and parental J774A.1 cells for 4 h infection (top: original magnification × 10,000, bottom: original magnification × 30,000). **(F)** Intracellular microbial killing assay with viable *V. vulnificus* in parental and NLRP3 knockout J774A.1 cells after infection with 2 MOI *V. vulnificus* for 4 h. The cells were treated with 100 μg/ml gentamycin for 30 min to kill the attached bacteria. Then, the cells were lysed, and live bacteria in cell lysates were counted after incubation on BHI blood plates. Data (not including RNA-Seq data) shown are representative of at least three experiments. **P < 0.01; ***P < 0.001 as determined by Student’s *t*-test. RNASeq data are representative of at least two experiments.

To understand how *V. vulnificus* causes inflammation and cell damage in macrophages, we performed RNA-Seq and transcriptomic analysis in J774A.1 cells before and after *V. vulnificus* infection. Compared with the infectious diseases gene pattern in the KEGG database, some genes responding to *V. vulnificus* infection were similar to those of 11 infectious diseases (*P* < 0.05), such as *tuberculosis*, *salmonella* infection, *legionellosis*, *leishmaniasis*, human cytomegalovirus (HCMV) infection, and influenza A infection (Figure 1A). Interestingly, as shown in Figure 1A, only one gene (IL-1β) that responded to *V. vulnificus* infection was overlapping among *tuberculosis*, *salmonella* infection, *legionellosis*, *leishmaniasis*, HCMV infection, measles, and influenza A. Furthermore, only six genes (*Nlrp3*, *Cd36*, *Ccl3*, etc.) overlapped in the core of the gene sets related to defense response, host response to the bacterium, innate immune response, IL-1β production, and regulation of the cellular metabolic process (Figure 1A). Next, we examined the expression of NLRP3 mRNA and protein in macrophages after *V. vulnificus* infection *in vitro* and *in vivo*. We found that NLRP3 mRNA expression was increased in Kupffer cells (KCs) from *V. vulnificus*-infected mice. Both NLRP3 mRNA expression and protein expression were elevated in bone marrow-derived macrophages (*BMMϕs*) and J774A.1 cells after *V. vulnificus* infection *in vitro* (Figures 1B,C). These data suggested that the expression of NLRP3 was enhanced after *V. vulnificus* infection.

To further investigate the essential role of NLRP3 in *V. vulnificus*-infected macrophages, we generated an NLRP3 knockout J774A.1 cell line using the CRISPR/Cas9 system. The J774A.1 cells were transfected with a plasmid expressing a single guide RNA (sgRNA) targeting the endogenous mouse *Nlrp3* exon two locus. The mutation caused a cleavage in the *Nlrp3* locus at exon 2. Successful targeting was detected after PCR amplification of a 500 bp amplicon, flanking the target region of *Nlrp3*, reannealing of the PCR product, and selectively digesting the mismatched heteroduplex fragments by T7EI. T7EI recognizes and cleaves DNA mismatches in heteroduplexes. Homozygous NLRP3 KO or wild-type DNA alone shows the full-length band (about 500 bp), whereas heterozygous editing of NLRP3 alone or homozygous NLRP3 KO mixed with wild-type DNA shows a cleaved band. As shown in Supplementary Figure S1A, only NLRP3 KO mixed wild-type DNA showed a cleaved band, indicating that the mutant NLRP3 J774A.1 cells (KO cell) were homozygous. This mutation was further confirmed by DNA sequencing, which revealed a 10 bp deletion in exon 2, resulting in a frameshift mutation (Supplementary Figure S1B) and complete loss of NLRP3 expression (Figure 1D).

NLRP3-deficient J774A.1 cells contained more *V. vulnificus* than parental J774A.1 cells as observed in electron microscopy analysis (Figure 1E). The majority of internalized *V. vulnificus* in parental J774A.1 cells was degraded in the phagosome vacuole (Figure 1E). By contrast, intact *V. vulnificus* was observed inside phagosomes in the NLRP3-deficient J774A.1 cells (Figure 1E). Furthermore, more viable *V. vulnificus* could be detected in NLRP3-deficient J774A.1 cells than in parental cells (Figure 1F). This result indicates that NLRP3 restricts the intracellular growth of *V. vulnificus* in the macrophages. However, these results still cannot rule out whether *V. vulnificus*-caused parental cell damage is sufficiently disrupted to allow penetration by antibiotic to kill intracellular *V. vulnificus*. Next, we determined the cell viability of parental and NLRP3-deficient J774A.1 cells before and after *V. vulnificus* infection by flow cytometry. As shown in Supplementary Figures S2A,B, there is no significant difference between phosphate-buffered saline (PBS) and *V. vulnificus*-infected parental and NLRP3-deficient J774A.1 cells. Together, these results suggest that the NLRP3 regulation in the killing of intracellular bacteria in the macrophage is not due to cell death.

### Impaired Phagosome Acidification and Phagolysosomal Fusion Cause Compromised Degradation of Internalized *V. vulnificus* in NLRP3-Deficient Macrophages

**FIGURE 2:**
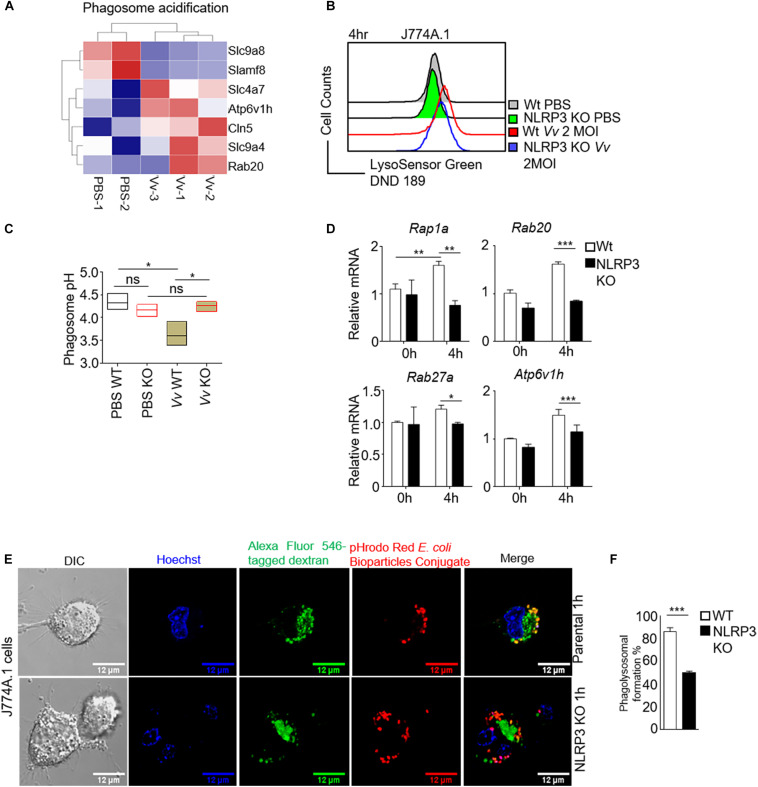
NLRP3 is crucial for regulation of phagosome acidification and phagolysosome formation in *V. vulnificus*-infected macrophages. **(A)** Heatmap of significantly enriched gene set in phagosome acidification from *V. vulnificus*-infected and uninfected J774A.1 cells (*P* < 0.05). **(B)** Phagosome acidification was detected by pH indicator dye LysoSensor Green DND-189 staining. After 4 h of infection with 2 MOI *V. vulnificus*, the cells were stained with 1 μM LysoSensor Green DND-189 and then subjected to flow cytometry analysis. **(C)** The phagosome pH in parental and NLRP3-deficient J774A.1 cells was measured by LysoSensor Yellow/Blue DND-160. Phagosome pH values were determined from calibration curves generated from permeabilized cells in various pH buffers. **(D)** RT-PCR analysis of mRNA expression of genes in phagosome maturation and acidification. **(E)** Confocal images are shown in phagolysosome formation. The Alexa Fluor 546 conjugated dextran was ingested by macrophage through endocytosis. The cells were cultured for 4 h to let the endosome develop to a mature lysosome. The pHrodo Red *E. coil* BioParticles conjugate uptake by the cells through phagocytosis. The phagolysosome formation is visualized by phagosome and lysosome fusion. Scale bar: 12 μM. **(F)** The bar figure shown is the percentage of the phagosome and lysosome fusion in NLRP3-deficient and parental J774A.1 cells after 2 MOI *V. vulnificus* infection. Data (not including RNA-Seq data) shown are representative of at least three experiments. *P < 0.05; **P < 0.01; ***P < 0.001 as determined by Student’s *t*-test. RNASeq data are representative of at least two experiments.

The elimination of bacteria by macrophages is a dynamic process involving bacteria internalization, phagosome formation, and phagosome and lysosome fusion (Kinchen and Ravichandran, 2008; Kim et al., 2019). The sufficient clearance of extracellular bacteria is dependent on both initial phagocytosis and functional phagolysosome (Buchmeier and Heffron, 1991; Wong et al., 2017). An acidic environment and the activation of pH-sensitive enzymes contribute to degrading the internalized bacteria in the bactericidal function of macrophages (Kinchen and Ravichandran, 2008; Sedlyarov et al., 2018). Our transcriptomic analysis in J774A.1 cells revealed that the phagosome acidification pathway was significantly upregulated after *V. vulnificus* infection (Figure 2A). To investigate whether impaired intracellular bacterial killing capacity upon NLRP3 loss was correlated with phagosome acidification damage. Lysosenor Green DND 189 staining (which measures the pH value of acidic organelles) was used to test phagosome acidification. We found that phagosomes containing *V. vulnificus* did not acidify sufficiently in NLRP3-deficient J774A.1 cells (Figure 2B). The phagosomes acidified to pH <4 after 4 h post-infection in parental J774A.1 cells (Figure 2C), whereas the phagosomes acidified to pH >4 in *V. vulnificus*-infected NLRP3 KO cell (Figure 2C). These results suggested that NLRP3 was required for maintaining sufficient phagosome acidification to clear the intracellular *V. vulnificus*. We also tested the mRNA expression of phagosome acidification-related genes, including *Rap1a*, *Rab20*, *Rab27a*, and *Atp6v1h.* The mRNA expression of these genes was significantly decreased in *V. vulnificus*-infected NLRP3-deficient J744A.1 cells (Figure 2D).

A new phagosome undergoes a process termed “maturation” by fusion and limited fission events with endosomes and lysosomes to generate an acidic and highly hydrolytic mature phagolysosome (Kinchen and Ravichandran, 2008). Phagocytosis and phagosome maturation eventually lead to progressive acidification of the phagolysosome. The low pH value in the mature phagolysosome is required for the ingestion and elimination of invading pathogens. Then, we determined whether losing NLRP3 caused a failure of phagolysosome formation. Endocytic trafficking to lysosomes in J774A.1 cells was examined by incubation of macrophages with Alexa Fluor 546 dextran for 2 h. The internalization of pHrodo *Escherichia coli* BioParticles by J774A.1 cells strongly suggested phagosomes. Phagolysosome formation, including the maturation of phagosomes fused with lysosomes, was determined by an analysis of the colocalization of dextran and pHrodo *E. coli* BioParticles. As shown in Figures 2E,F, in the parental J774A.1 cells, dextran staining was partially colocalizing with internalized pHrodo *E. coli* BioParticles, indicating the fusion of lysosome and phagosome. However, there is almost no colocalization of dextran with pHrodo *E. coli* BioParticles in NLRP3-deficient J774A.1 cells, which meant a blockage in phagolysosome formation. Together, our results suggested a disruption of phagosome acidification and Gram-negative bacteria-mediated phagolysosome formation with NLRP3 deficiency in macrophages.

### mTORC1 and NLRP3 Reciprocally Regulate *V. vulnificus*-Infected Macrophage Bactericidal Activity

**FIGURE 3:**
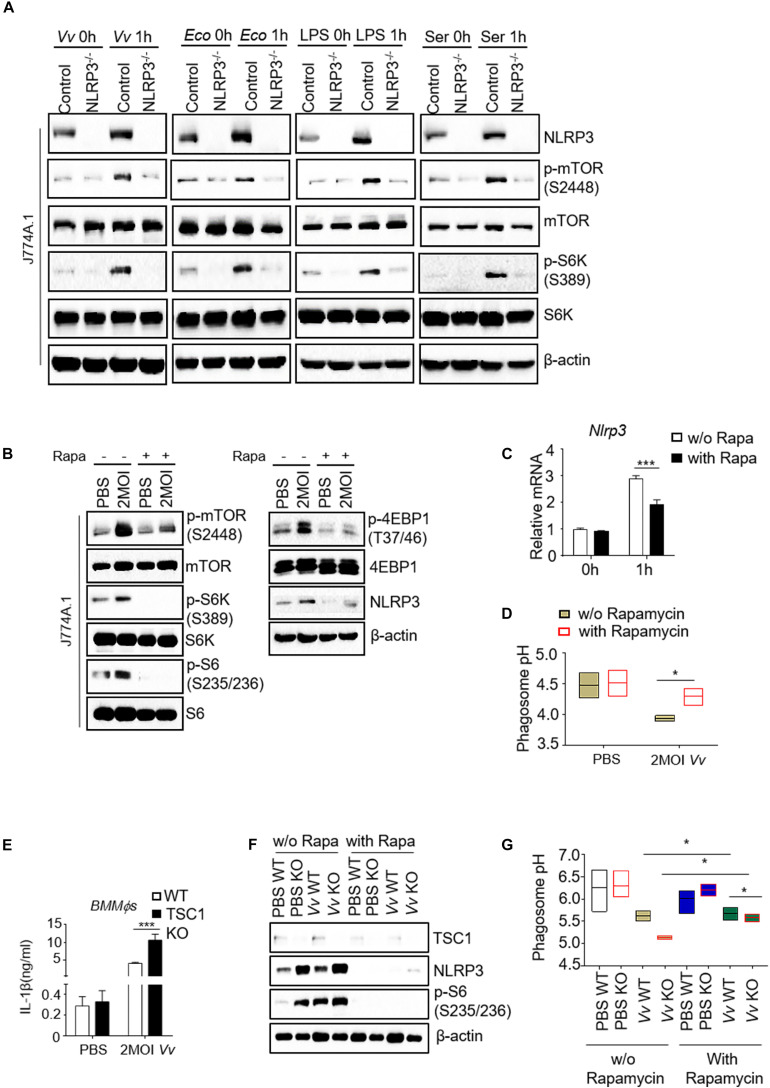
Reciprocal regulation of NLRP3 and mTORC1 impairs phagosome acidification in *V. vulnificus*-infected macrophages. **(A)** Western blot analysis of mTOR signaling in parental and NLRP3 knockout J774A.1 cells upon different stimulations. **(B)** Western blot analysis of mTOR signaling and NLRP3 expression in J774A.1 cells with indicated treatments. **(C)** RT-PCR analysis of *Nlrp3* mRNA expression in the J774A.1 cells with or without rapamycin after *V. vulnificus* infection. **(D)** pH values of phagosomes in the indicated J774A.1 cells. **(E)** IL-1β secretion in the supernatant of WT and TSC1 KO *BMMϕs* after *V. vulnificus* infection for 4 h. **(F)** Western blot analysis of mTOR signaling and NLRP3 expression in 2 MOI *V. vulnificus*-infected or -uninfected wild-type and TSC1 KO *BMMϕs* with or without 100 nM rapamycin. **(G)** Phagosome pH in WT and TSC1 KO *BMMϕs* was measured by LysoSensor Yellow/Blue DND-160. Data shown are representative of at least three experiments. Rapa, rapamycin; WT, C57BL/6J wild-type mice; TSC1 KO, TSC1^flox/flox^ LysM-Cre^+^ mice. *P < 0.05; ***P < 0.001 as determined by Student’s t-test.

mTOR is a master kinase that regulates lysosome structure and function in lysosomal biogenesis, distribution, and activity (Puertollano, 2014). mTOR also localizes to the lysosomal membrane, which is a crucial regulator for the cellular metabolism homeostasis (Wang et al., 2017; Rabanal-Ruiz and Korolchuk, 2018). To address this issue, we analyzed mTORC1 signaling activity in NLRP3-deficient and parental J774A.1 cells with various stimulation. Incubated with *V. vulnificus*, *E. coli*, lipopolysaccharide (LPS), or serum, the NLRP3-deficient J774A.1 cells showed comparable increased phosphorylation levels in both mTOR and S6K1 (Figure 3A). Moreover, lysates of rapamycin-treated macrophages were analyzed for mTORC1 signaling. The protein phosphorylation level of mTOR, S6K, S6, and 4E-BP1 in *V. vulnificus*-infected J774A.1 cells treated with rapamycin was dramatically decreased compared with those cells without rapamycin (Figure 3B). Besides, we observed an additional inhibitory effect on the expression of NLRP3 in both mRNA and protein levels in J774A.1 cells after *V. vulnificus* infection (Figures 3B,C). To avoid the NLRP3 KO clone could result from just clone to clone variation rather than loss of NLRP3. We also detected other clones of NLRP3 KO J774A.1 cells (Supplementary Figure S3A). The mTOR signaling was also impaired in NLRP3 KO cells (Supplementary Figure S3B). These data demonstrated that *V. vulnificus*-induced mTORC1 activation and NLRP3 expression could be inhibited by rapamycin. Besides, we observed a significantly elevated pH value in rapamycin-treated parental J774A.1 cells (Figure 3D).

To further address the relationship between mTORC1 overactivation and NLRP3 expression during *V. vulnificus* infection, we infected the *BMMϕs* from Tuberous Sclerosis Complex 1 (TSC1)^flox/flox^ LysM-Cre^+^ mice (TSC1 KO) and C57BL/6J (wild-type, WT) mice with 2 multiplicity of infection (MOI) *V. vulnificus.* Infected TSC1 KO *BMMϕs* increased the production of mature IL-1β in response to *V. vulnificus* challenge (Figure 3E). As shown in Figure 3E, S6 phosphorylation increased in TSC1 KO *BMMϕs*, indicating that mTORC1 signaling overactivated. S6 phosphorylation in both wild-type and TSC1 KO *BMMϕs* was increased after *V. vulnificus* infection (Figure 3F). However, NLRP3 expression increased in *V. vulnificus*-uninfected TSC1 KO *BMMϕs* and *V. vulnificus*-infected WT *BMMϕs*, indicating that mTORC1 overactivation could enhance NLRP3 expression, which could be reversed by rapamycin treatment (Figure 3F). Besides, the pH value in *V. vulnificus*-infected TSC1-deficient *BMMϕs* was much lower than that in WT *BMMϕs* (Figure 3G). However, by comparing with J774A.1 cells (Figure 3D), the pH *BMMϕs* was much higher, suggesting that the pH value could be variable in different cell lines. These results indicated that there was crosstalk between NLRP3 and mTORC1 in phagosome acidification.

### Endogenous NLRP3 Interacts With mTOR in Macrophages

**FIGURE 4:**
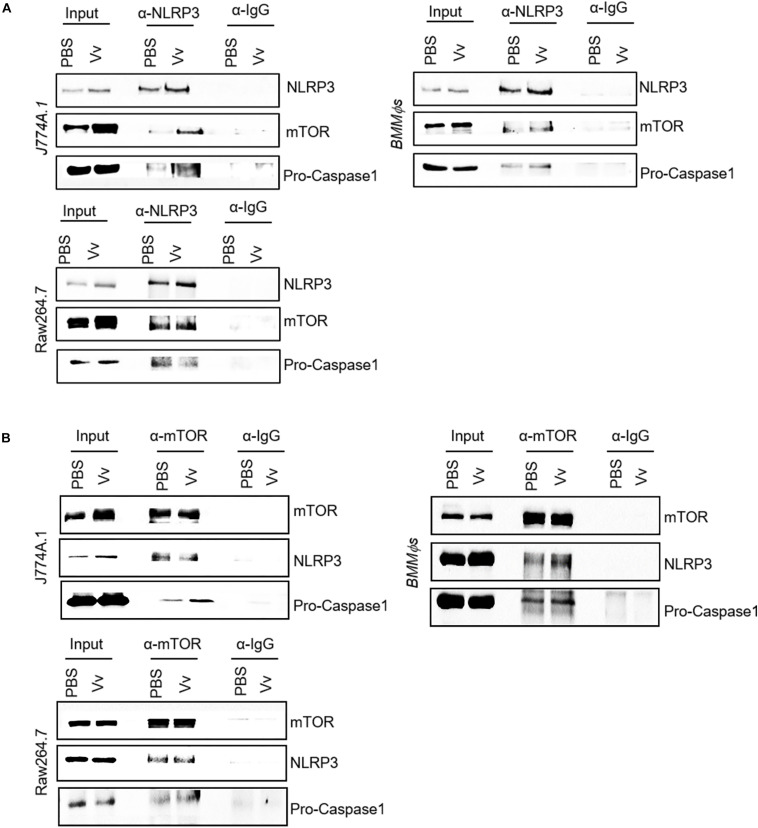
Interaction between NLRP3 and mTOR in murine macrophages. **(A,B)** Whole-cell lysates from PBS-treated or *V. vulnificus*-infected J774A.1 cells, Raw264.7 cells, and *BMMϕs* were immunoprecipitated with NLRP3 antibody **(A)** or mTOR antibody **(B)**, and Western blots were performed with antibodies against NLRP3, mTOR, and pro-caspase-1. Data shown are representative of at least three experiments.

To investigate the underlying mechanism of NLRP3 and mTOR reciprocal regulation of phagosome acidification and host bactericidal activity in macrophages during *V. vulnificus* infection, we conducted an immunoprecipitation assay in *V. vulnificus*-infected or -uninfected WT *BMMϕs*, J774A.1 cells, and Raw264.7 cells using anti-NLRP3 or anti-mTOR monoclonal antibody. We found that endogenous NLRP3 interacted with mTOR not only in J774A.1 cells but also in Raw264.7 cells and WT *BMMϕs* (Figure 4A). The *V. vulnificus* infection could enhance the interaction between NLRP3 and mTOR by the upregulation of NLRP3 expression, indicating that the protein–protein interaction was likely affected by *V. vulnificus* infection (Figure 4A). In the total cell lysate (input), the NLRP3 band slightly shifted after immunoprecipitation (Figure 4A). The band from SDS-PAGE lanes was used to confirm whether it was NLRP3 by mass spectrometry (liquid chromatography with tandem mass spectrometry, LC–MS/MS). The LC–MS/MS results confirmed that the hit with the highest score of peptides was NLRP3 (Supplementary Figure S4 and Supplementary Table S2). Moreover, we used an anti-mTOR monoclonal antibody to pull down endogenous mTOR. The blots showed that mTOR interacted with NLRP3 in J774A.1 cells, Raw264.7 cells, and WT *BMMϕs* (Figure 4B). Additionally, we observed that the interaction between NLRP3 and NLRP3 inflammasome components (e.g., pro-caspase-1) or mTOR and pro-caspase-1 simultaneously existed (Figures 4A,B), which indicated that pro-caspase-1 might be constitutively associated with NLRP3. Collectively, these data highlight mTOR as a novel component of the NLRP3 inflammasome, and the reciprocal regulation between mTORC1 and NLRP3 is likely *via* protein–protein interaction. However, the role of mTOR in association with NLRP3 is still not currently understood.

## Discussion

Although much of the recent work has linked the pathogenicity of *V. vulnificus* infection to innate and adaptive immune response, inflammasome activation, cytotoxicity of virulence factors (e.g., cell death and inflammation), and host iron metabolism, little is known about how host phagocytes eliminate pathogens encountering *V. vulnificus* (Jones and Oliver, 2009; Toma et al., 2010; Pajuelo et al., 2014; Arezes et al., 2015; Lee et al., 2017; Xie et al., 2017; Baker-Austin and Oliver, 2018). Our study made several advances in illuminating the crosstalk between NLRP3 and mTOR in the regulation of bactericidal activity, including phagosome acidification, phagolysosome formation, and intracellular bacterial clearance in macrophage during *V. vulnificus* infection. Our results suggested a novel mechanism about the reciprocal regulation of NLRP3 and mTORC1 signaling in phagolysosome-mediated intracellular microbial killing in *V. vulnificus*-infected macrophages.

NLRP3 is involved in acidification of phagosomes bearing Gram-positive but not Gram-negative organisms (Sokolovska et al., 2013). However, we found that Gram-negative bacteria, such as *V. vulnificus*, also boost NLRP3 expression. The absence of NLRP3 could cause insufficient intracellular bacterial clearance *via* impaired lysosomal function, phagosome acidification, and phagolysosome formation. Our results bolster a recent study (Sokolovska et al., 2013) that links caspase-1 to regulate phagosome acidification and phagolysosome formation. By deletion of NLRP3, the phagosome failed to rapidly acidify. The insufficient phagosome acidification allows the sustained survival of *V. vulnificus* in the phagocytes and results in the delay of invoked microbial degradation. Here, we show that NLRP3 deficiency in macrophages impairs the expression of various genes linked to phagosome acidification and maturation, including *Rap1a*, *Rab27a*, *Rab20*, and *Atp6v1h*. Previous studies have shown that a GTPase family protein, Rap1A, was localized to late endosome/phagosome compartments in J774 macrophages by colocalization with LAMP2, which may be involved in late endocytic/phagocytic processes (Chung et al., 2008). Rab27a also belongs to the family of small GTPase, which is localized on dendritic cell–phagosomes and is implicated in the regulation of phagosome acidification in dendritic cells (Jancic et al., 2007). Rab20 is enriched on the phagosome, which is likely essential for phagosome maturation. Phagosome maturation includes the acquisition of vacuolar ATPase, a proton pump involved in the maintenance of intracellular organellar acidification (Kinchen and Ravichandran, 2008; Russell et al., 2009). A mature form of phagosome is crucial for phagolysosomal fusion, and phagolysosomes display sufficient antimicrobial capacity. Thus, the V-type proton ATPase complex, such as *Atp6ap1*, *Atp6v1a*, and *Atp6v1h*, is thought to be a dynamic marker for phagolysosomal fusion (Xiong and Zhu, 2016). Together, our results extend the previous findings from Sokolovska et al. (2013). Our study shows that NLRP3 is likely essential for operating the phagosome and lysosome fusion processes through small GTPase and V-type proton ATPase complex genes.

Moreover, our results show that rapamycin completely blocks the phosphorylation of S6 in *V. vulnificus*-infected macrophages. The pH in *V. vulnificus*-infected TSC1 KO macrophages is still lower than that in WT macrophages in the presence of rapamycin. It suggests that TSC1 likely regulates pH in *V. vulnificus*-infected macrophages in an mTOR-independent manner. Whether this mTOR-independent manner is involved in regulating phagosome acidification still needs to be determined in the future. Recent studies have also revealed that mTORC1 activity is important for phagosomes to undergo late maturation (Krajcovic et al., 2013). Additionally, the activation of NLRP3 inflammasome leads to the leakage of lysosomal contents into the cytosol after phagocytosis of particulates (Amaral et al., 2018). Alternatively, the release of reactive oxygen species (ROS) into the cytosol during lysosomal membrane permeabilization also could trigger NLRP3 inflammasome activation (Heid et al., 2013). Thus, the mechanisms underlying NLRP3 inflammasome, mTOR activity, and phagosome/lysosome in defense of Gram-negative bacterial infection still require clarification. Our results found a protein–protein interaction between NLRP3 and mTOR in macrophages, and the absence of NLRP3 significantly inhibits mTORC1 activation. Whereas, increasing mTORC1 activation by deletion of TSC1 in macrophages resulted in enhanced NLRP3 expression, which could be reversed by rapamycin. Besides, inhibited mTORC1 activation by rapamycin also reduced the NLRP3 protein expression, following increased phagosome pH. Our results indicated that mTOR kinase activity was likely involved in NLRP3 stability/post-translation through protein–protein interaction. Cosin-Roger et al. (2017) also reported NLRP3 as a novel binding partner of mTOR in HT-29 cells regulated by hypoxia. In our results in correspondence with their results, we found that the physical interaction of NLRP3 and mTOR exists in both macrophage cell lines (J774A.1 cells and RAW264.7 cells) and primary macrophages (*BMMϕs*). This interaction plays an essential role against *V. vulnificus* infection. Whereas NLRP3 lacks a caspase recruitment domain (CARD), it cannot recruit pro-caspase-1 except in the presence of the adaptor molecule apoptosis-associated speck-like protein containing a CARD (ASC) (Davis et al., 2011). Interestingly, RAW264.7 cells do not have the ASC adapter, indicating that pro-caspase-1 would have to directly associate with NLRP3 or another complex component (e.g., mTOR), not through the ASC adapter. Future studies will be needed to clarify whether the interaction of NLRP3 and mTOR in macrophages plays a crucial role in pathogen clearance *in vivo*.

Li et al. (2018) have found that mTORC1/2 inhibitor INK128 decreases both mRNA and protein levels of NLRP3 in LPS/ATP and LPS/nigericin-stimulated murine *BMMϕs*. Additionally, Nakahira’s group has reported that Torin 1 suppresses mTOR1/2 activity, which inhibits NLRP3 inflammasome activation through hexokinase-1 (HK-1) glycolysis in macrophages (Moon et al., 2015). These previous studies indicate that mTOR kinase activity is likely involved in NLRP3 mRNA transcription and protein expression. We found that the inhibition of mTORC1 activation by rapamycin could suppress *V. vulnificus*-induced NLRP3 expression in both mRNA and protein levels. Our results not only are consistent with these previous studies but also strengthen the role of mTORC1 in the regulation of NLRP3 expression. Moreover, our results provide new clues for the relationship between mTOR and NLRP3 through protein–protein interaction in the macrophages. Whether mTOR-mediated HK1-dependent glycolysis is involved in the regulation of *V. vulnificus*-induced NLRP3 expression is still needed to be determined in the future.

Overall, our results indicate that the reciprocal regulation of mTOR and NLRP3 is important for intracellular *V. vulnificus* clearance in macrophages. Activated mTOR signaling and upregulated NLRP3 allow macrophages to limit internalized microbes in the host cells and establish efficient phagolysosomal function in defense of *V. vulnificus* infection. Whether the NLRP3–mTOR reciprocal regulation plays a similar role in the modulation of host defense to other Gram-negative bacteria or intracellular microbe infection remains to be addressed.

## Materials and Methods

### Mice

Six- to eight-week-old C57BL/6J mice were purchased from the Chinese Academy of Sciences, Shanghai Laboratory Animal Center (SLAC). Tsc1 flox mice and Lysozyme Cre transgenic mice were obtained from the Jackson Laboratory. Tsc1 flox mice were bred with Lysozyme Cre mice to induce TSC1 deletion in myeloid cells. All the mice were maintained and used in accordance with the animal experimental guidelines set by the Wenzhou Medical University Animal Care and Use Committee.

### Bacterial Strains and Cell Culture

L-929 cells and J774A.1 cells were purchased from the Cell Bank of Type Culture Collection of Chinese Academy of Sciences in Shanghai. All the cells were cultured in RPMI 1640 containing 10% heat-inactivated fetal bovine serum (FBS, Ausvin) and penicillin–streptomycin (50 IU/ml and 50 mg/ml, Beyotime). The China General Microbiological Culture Collection Center provided the *V. vulnificus* CGMCC 1.1758 strain. We grew *V. vulnificus* at 37°C in brain heart infusion (BHI) broth or on the BHI rabbit blood agar plate. The procedures of *V. vulnificus* that were used in this study were followed from the standard biological hazards at Biosafety Level 2 and the safety procedures of Wenzhou Medical University Laboratory Safety Department.

### Generation of Bone Marrow-Derived Macrophages

The bone marrow cells were isolated from femurs and tibiae and cultured with a 10% L-929 cell culture medium, as described previously (Pan et al., 2012). After 3 days of culturing at 37°C in a CO_2_ incubator, non-adherent cells were collected and transferred into new Petri dishes with a freshly conditioned medium. After another 4 days of culture, the *BMMϕs* were ready for experimentation. Flow cytometry was used to analyze the F4/80^+^CD11b^+^ cell purity. Over 90% of the cells were F4/80^+^CD11b^+^ cells.

### Bacterial Treatment of *BMMϕs* and J774A.1 Cells *in vitro*

*BMMϕs* and J774A.1 cells were plated in 35 mm dishes, and then *V. vulnificus* was added to the cells at the indicated MOI. The supernatant was collected at the indicated time for cytokine quantification. The infected cells were used for flow cytometry analysis, confocal microscopy analysis, quantitative reverse transcription PCR (RT-qPCR) analysis, and Western blot analysis.

### Phagosome Acidification

To measure phagosome acidification, the cells were treated with 1 μM LysoSensor Green DND-189 for 30 min at 37°C and then were analyzed by flow cytometry. Additionally, to obtain a pH standard curve, the cells were loaded with LysoSensor Yellow/Blue DND-160 for 5 min at 37°C; the lysosomal pH was equalized by incubating cells for 5 min at 37°C before incubating in a calibration buffer (125 mM KCl, 25 mM NaCl, 10 μM monensin, and 25 mM 4-morpholineethanesulfonic acid hydrate (MES) with the pH adjusted to either 3.5, 4.0, 4.5, 5.0, 5.5, 6.0, or 6.5, respectively). Then, the cells were incubated at the same time points that in generating the pH calibration curve and washed twice with PBS. Data were collected with a microplate reader by excitation at 440 and 540 nm.

### Phagolysosome Formation

The phagolysosome formation was determined as previously described (Baker et al., 2015). Briefly, the cells were incubated with Alexa Fluor 488-tagged dextran (250 μg/ml) for 1 h after 1 μg/ml Hoechst 33342 staining (Beyotime). The cells were then washed three times and incubated in RPMI 1640 medium for 3 h to allow lysosome translocation of dextran. The cells were subjected to incubation with pHrodo Red *E. coli* BioParticles for 1 h. Data were collected using a Nikon A1 confocal microscope.

### Transcriptome Profiling

J774A.1 cells with 4 h *V. vulnificus* infection or PBS treatment were used for transcriptome sequencing by Annoroad Co. Significantly differentially expressed genes were identified when we compared the normalized reads count between *V. vulnificus* and PBS groups with *P* < 0.05 and |Log2FoldChange| > 0.263. The significance of the gene ontology term enrichment was estimated using Fisher’s Exact Test (*P*-value).

### Real-Time qPCR Analysis

Total RNA was extracted from sorted KCs, *BMMϕs*, and J774A.1 cells by TRIzol reagent (Omega Bio-Tek). cDNA was synthesized using the FastQuant RT Kit (TIANGEN). Real-time qPCR was performed as previously described (Xie et al., 2012). Target mRNA expression was normalized with β-actin and then was calculated using the 2^–ΔΔCT^ method. Primers were shown in Supplementary Table S1.

### Western Blot Analysis

Cell lysates preparation and Western blot analysis were performed by following the previous protocol (Xie et al., 2012). Antibodies against p-mTOR, mTOR, NLRP3, p-S6K, p-4EBP1, p-S6, β-actin, S6K, S6, and 4EBP1 were purchased from Cell Signaling Technology (CST).

### Generation of an NLRP3-Deficient J774A.1 Cell Line

Murine *Nlrp3* guide RNA was designed using an online tool^1^. By following the previously described protocol (Ran et al., 2013), the annealed oligo (Forward, 5'-CAC CGA CAT TCC TCT ATG GTA TGC C-3'; Reverse, 5'-AAA CGG CAT ACC ATA GAG GAA TGT C-3') was cloned into pSpCas9 (BB)-2A-GFP (PX458) vector (Addgene) to generate PX458-NLRP3 plasmid. Then, PX458-NLRP3 plasmid was transfected into J774A.1 cells using the QuickShuttle-Superfast Transfection Kit (Biodragon Immunotech) following the manufacturer's protocol.

### Flow Cytometry

Fluorochrome–conjugated anti–CD11b and anti–F4/80 were purchased from Biolegend. Cell death was identified using a violet Live/Dead Kit or 7AAD staining (Invitrogen). The cells were stained with fluorescence conjugated antibodies in 2% (vol/vol) FBS in PBS at 4°C for 30 min. Data were collected using a BD FACSAria II and analyzed using the FlowJo software. CD11b^int^F4/80^+^ KCs from C57BL/6J mice were sorted using a BD FACSAria II sorter.

### Electron Microscopy

Electron microscopic analysis was performed as previously described (Lee et al., 2017). Briefly, the cells were washed with PBS and harvested into a 1.5 ml Eppendorf tube. The cells were then fixed with 2.5% glutaraldehyde, post-fixed in 1% osmium tetroxide and 1% potassium, and embedded in Quetol 812. Ultrathin sections were stained with uranyl acetate and lead nitrate. Images were observed and acquired using an H-7500 Hitachi transmission electron microscope.

### Immunoprecipitation

J774A.1 cells, RAW264.7 cells, and *BMMϕs* were infected with 2 MOI *V. vulnificus*. The cells were lysed for total protein using Pierce^TM^ IP Lysis Buffer (Thermo Fisher Scientific) containing protease and phosphatase inhibitors (Sigma). In total, 500 μg cell lysate was incubated with 6 μl anti-mTOR antibody (CST) or anti-NLRP3 antibody (CST) or 2 μl anti-rabbit IgG (CST) overnight at 4°C to form the complex. The immunoprecipitation assay was performed using the Magnetic IP Kit (Thermo Fisher Scientific) according to the manufacturer’s protocol. Briefly, the antigen–antibody mixture was incubated with prewashed magnetic beads for 1 h and washed with wash buffer twice. Then, the beads were collected using a magnetic stand, eluted in a low-pH elution buffer, and then used for Western blot analysis.

### Intracellular Bacterial Growth

The cells were infected with 2 MOI *V. vulnificus* for 4 h and washed with PBS. The supernatant was diluted and plated on blood BHI plates for 12 h. Then, 100 μg/ml gentamicin was added to kill extracellular bacteria. After 30 min, the cells were washed twice, treated with 0.1% Triton X-100, and plated on blood BHI plates for 12 h at 37°C for a bacterial count.

### Statistical Analysis

Data were presented as mean ± SEM and analyzed for statistical differences using the Prism 6.01/GraphPad software. Statistical significance was analyzed using the Student’s *t*-test. *P*-values less than 0.05 were considered significant.

## Data Availability Statement

The raw data supporting the conclusions of this article will be made available by the authors, without undue reservation, to any qualified researcher.

## Ethics Statement

The animal study was reviewed and approved by the Wenzhou Medical University Animal Care and Use Committee.

## Author Contributions

D-LX, Y-LL, and YZ conceived and designed the experiments. D-LX, Y-LL, YZ, X-HH, YM, M-MZ, NC, M-NH, and L-YW analyzed the data. D-LX, YZ, X-HH, YM, M-MZ, NC, M-NH, and L-YW conducted the experiments. D-LX and X-HH wrote the manuscript. All authors contributed to the article and approved the submitted version.

## Conflict of Interest

The authors declare that the research was conducted in the absence of any commercial or financial relationships that could be construed as a potential conflict of interest.

## Footnotes

1 http://crispr.mit.edu
