# Supplementary material for: NLRP3 and mTOR Reciprocally Regulate Macrophage Phagolysosome Formation and Acidification Against Vibrio vulnificus Infection
Source: Front Cell Dev Biol. 2020 Oct 8;8:587961. doi: 10.3389/fcell.2020.587961 (PMC7578225; doi:10.3389/fcell.2020.587961)
Supplement: Supplementary file 1 [file Table_1.DOCX]

Supplementary Material

# Supplementary Figures and Tables

## Supplementary Figures


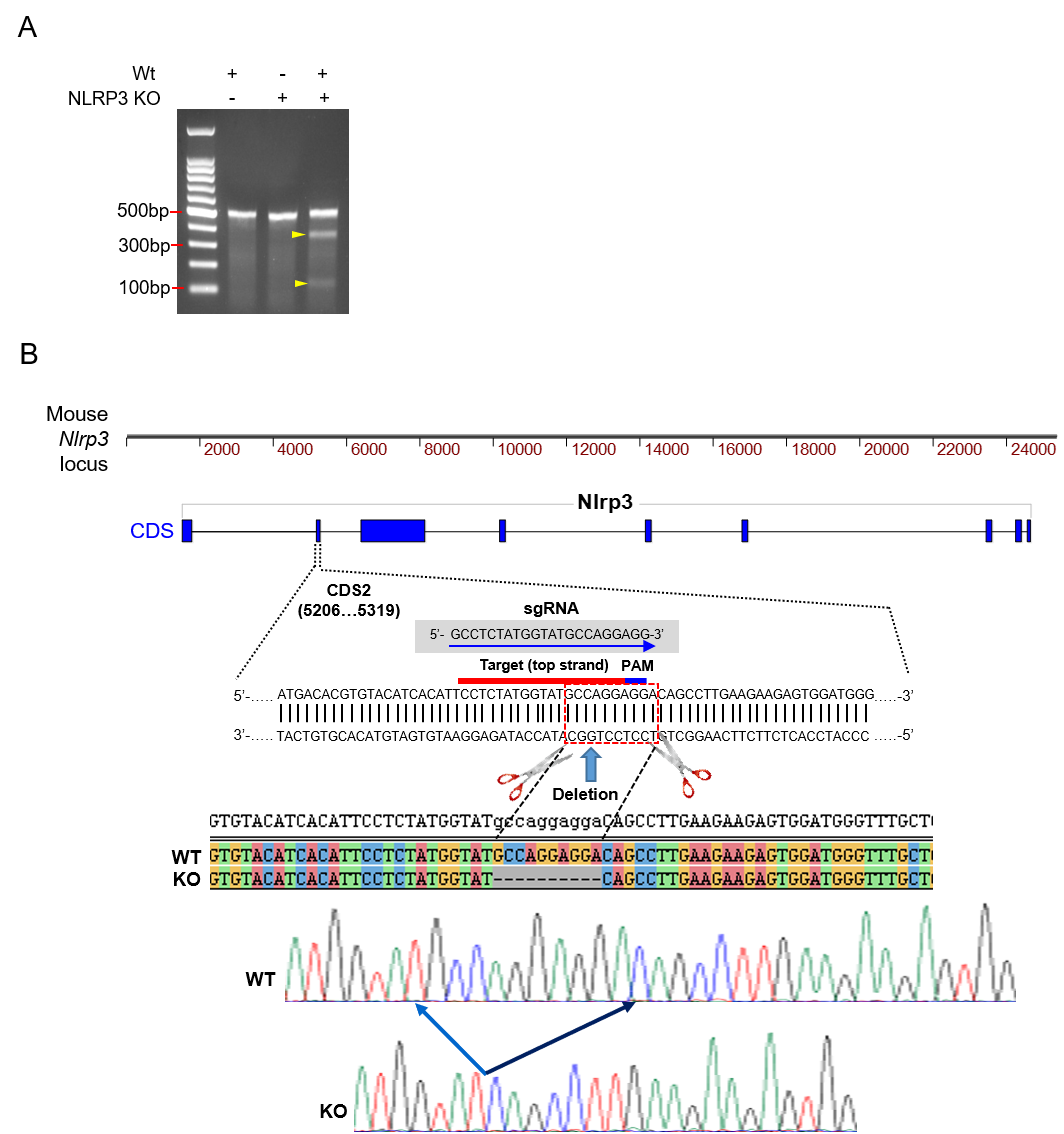


**Supplementary Figure 1.** **Generation of NLRP3 knockout J774A.1 cell line using CRISPR/Cas9 gene editing system. (A)** Identification of *Nlrp3* gene biallelic deletion clones by T7EI digestion assay. **(B)** The schematic of gene editing with CRISPR-Cas9 to generate NLRP3 deficient J774A.1 cells.


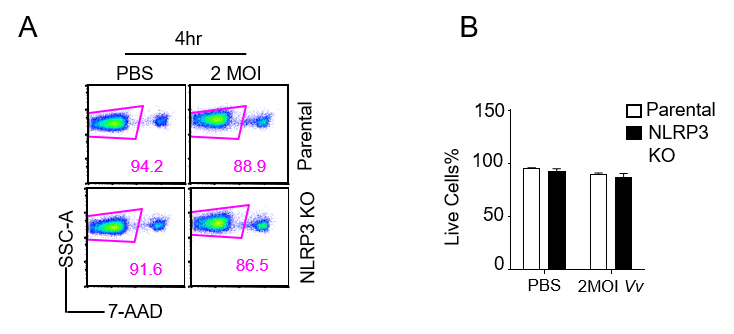


**Supplementary Figure 2.** **The viability of parental and NLRP3 KO J774A.1 cells with or without *V. vulnificus* infection. (A)** The representative dot blots show the cell viability by flow cytometry analysis. The live cells were gated on 7-AAD negative cells. **(B)** The bar figure shows means ± SEM of percentage of live cell with indicated treatment from three experiments.


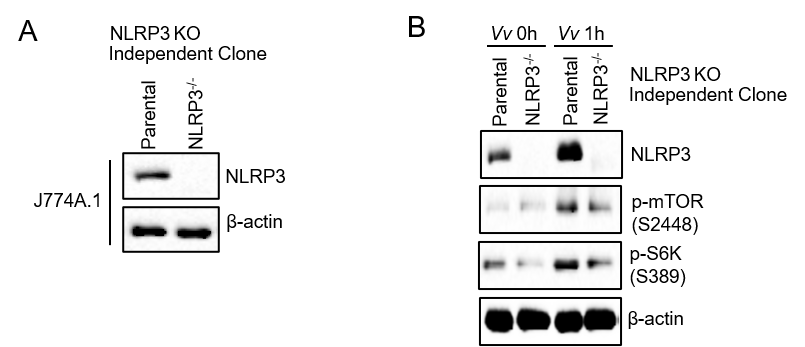


**Supplementary Figure 3. The mTOR signaling in another clone of NLRP3 KO J774A.1 cells. (A)** Identification of the NLRP3 expression in biallelic deletion clones by western blots. **(B)** Western blot analysis of mTOR signaling and NLRP3 expression in J774A.1 cells with indicated treatments. Data shown are representative of at least three experiments.


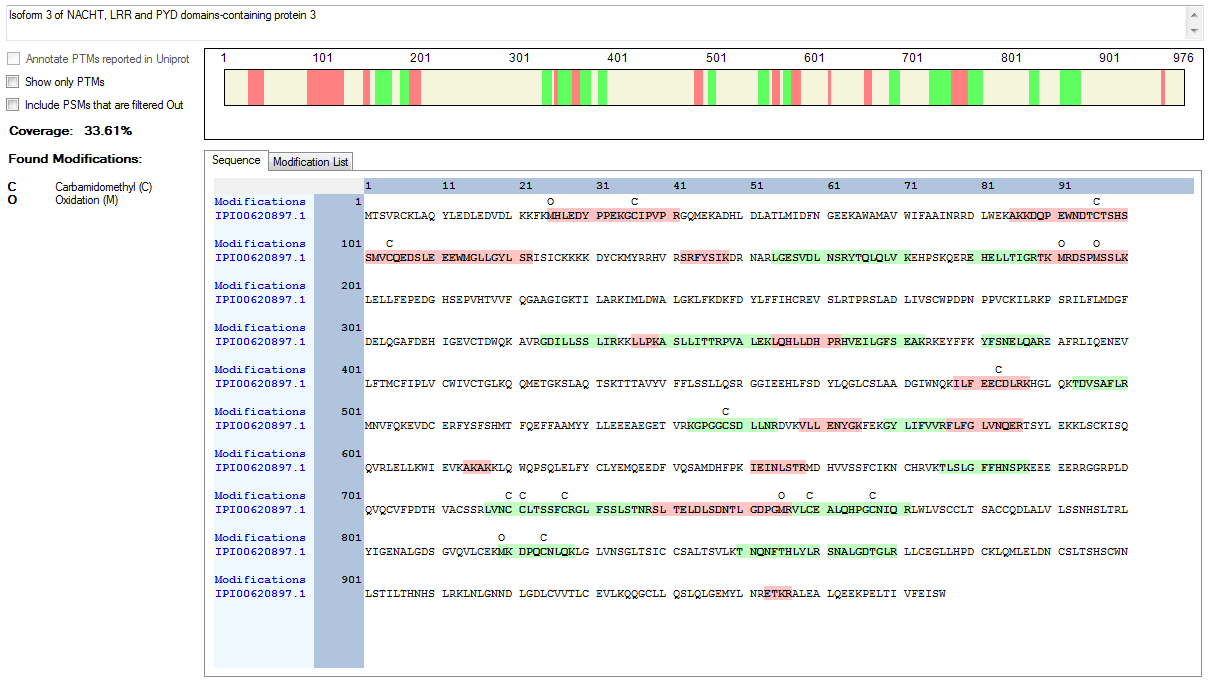


**Supplementary Figure 4. Identification of immunoprecipitated NLRP3 from J774A.1 cells by LC-MS/MS analysis.** The sample from immunoprecipitated NLRP3 band in SDS-PAGE gel was prepared and subjected to LC-MS/MS analysis.

## Supplementary tables

| **Supplementary Table 1. Primers used in this study** | |
| --- | --- |
| Primer | Sequence (5’–3’) |
| *Rap1a* F | GTAGTCCTTGGTTCAGGAGGC |
| *Rap1a* R | CAGGATCTCCAGCATGCACT |
| *Rab20* F | CGCCTTCTACCTGAAGCAGT |
| *Rab20* R | CTGTGGGTGGTTCACATCGT |
| *Rab27a* F | CCAGAGGGCAGTGAAAGAGG |
| *Rab27a R* | TCAGGTCCAGGAGCATCTCA |
| *Atp6v1h* F | CCGAGGCTATCCAGGTCTGT |
| *Atp6v1h* R | GTTGGCACGAACTTCAGCAG |
| *Nlrp3* F | CGAGACCTCTGGGAAAAAGCT |
| *Nlrp3* R | GCTGAGCAGCTGCAAACGAC |
| *β-actin* F | GGCTGTATTCCCCTCCATCG |
| *β-actin* R | CCAGTTGGTAACAATGCCATGT |

| **Supplementary Table 2. The results of NLRP3 identification by LC-MS/MS** | | | |
| --- | --- | --- | --- |
| **ACC** | **DESC** | **SCORE** | **MASS** |
| NALP3_MOUSE | NACHT, LRR and PYD domains-containing protein 3 | 573 | 120935 |
| MYO1C_MOUSE | Unconventional myosin-Ic | 485 | 122723 |
| SNPC4_MOUSE | snRNA-activating protein complex subunit 4 | 41 | 148348 |
| FYCO1_MOUSE | FYVE and coiled-coil domain-containing protein 1 | 37 | 164060 |
| EF2_MOUSE | Elongation factor 2 | 143 | 96222 |
| MYO1G_MOUSE | Unconventional myosin-Ig | 119 | 118123 |
| MYO1D_MOUSE | Unconventional myosin-Id | 84 | 116806 |
| HNRPU_MOUSE | Heterogeneous nuclear ribonucleoprotein U | 88 | 88661 |
| ITB1_MOUSE | Integrin beta-1 | 74 | 91424 |
| AT2A1_MOUSE | Sarcoplasmic/endoplasmic reticulum calcium ATPase 1 | 42 | 110723 |
| UBP8_MOUSE | Ubiquitin carboxyl-terminal hydrolase 8 | 35 | 123447 |
| COBL_MOUSE | Protein cordon-bleu | 35 | 144518 |
